# Cytosolic 5′-Nucleotidase II Silencing in a Human Lung Carcinoma Cell Line Opposes Cancer Phenotype with a Concomitant Increase in p53 Phosphorylation

**DOI:** 10.3390/ijms19072115

**Published:** 2018-07-20

**Authors:** Rossana Pesi, Edoardo Petrotto, Laura Colombaioni, Simone Allegrini, Mercedes Garcia-Gil, Marcella Camici, Lars Petter Jordheim, Maria Grazia Tozzi

**Affiliations:** 1 Unità di Biochimica, Dipartimento di Biologia, Università di Pisa, Via San Zeno 51, 56127 Pisa, Italy; rossana.pesi@unipi.it (R.P.); edoardo.petrotto@gmail.com (E.P.); simone.allegrini@unipi.it (S.A.); marcella.camici@unipi.it (M.C.); 2 Istituto di Neuroscienze, CNR, Via Giuseppe Moruzzi 1, 56124 Pisa, Italy; laura.colombaioni@in.cnr.it; 3 Unità Fisiologia Generale, Dipartimento di Biologia, Università di Pisa, Via San Zeno 31, 56127 Pisa, Italy; mercedes.garcia@unipi.it (M.G.-G.); 4 Université de Lyon, Université Claude Bernard Lyon 1, INSERM 1052, CNRS 5286, Centre Léon Bérard, Centre de Recherche en Cancérologie de Lyon, Lyon 69008, France; lars-petter.jordheim@univ-lyon1.fr

**Keywords:** Cytosolic 5′-nucleotidase, p53, 2-deoxyglucose, human lung carcinoma, cell proliferation

## Abstract

Purine homeostasis is maintained by a purine cycle in which the regulated member is a cytosolic 5′-nucleotidase II (cN-II) hydrolyzing IMP and GMP. Its expression is particularly high in proliferating cells, indeed high cN-II activity or expression in hematological malignancy has been associated to poor prognosis and chemoresistance. Therefore, a strong interest has grown in developing cN-II inhibitors, as potential drugs alone or in combination with other compounds. As a model to study the effect of cN-II inhibition we utilized a lung carcinoma cell line (A549) in which the enzyme was partially silenced and its low activity conformation was stabilized through incubation with 2-deoxyglucose. We measured nucleotide content, reduced glutathione, activities of enzymes involved in glycolysis and Krebs cycle, protein synthesis, mitochondrial function, cellular proliferation, migration and viability. Our results demonstrate that high cN-II expression is associated with a glycolytic, highly proliferating phenotype, while silencing causes a reduction of proliferation, protein synthesis and migration ability, and an increase of oxidative performances. Similar results were obtained in a human astrocytoma cell line. Moreover, we demonstrate that cN-II silencing is concomitant with p53 phosphorylation, suggesting a possible involvement of this pathway in mediating some of cN-II roles in cancer cell biology.

## 1. Introduction

**Scheme 1:**
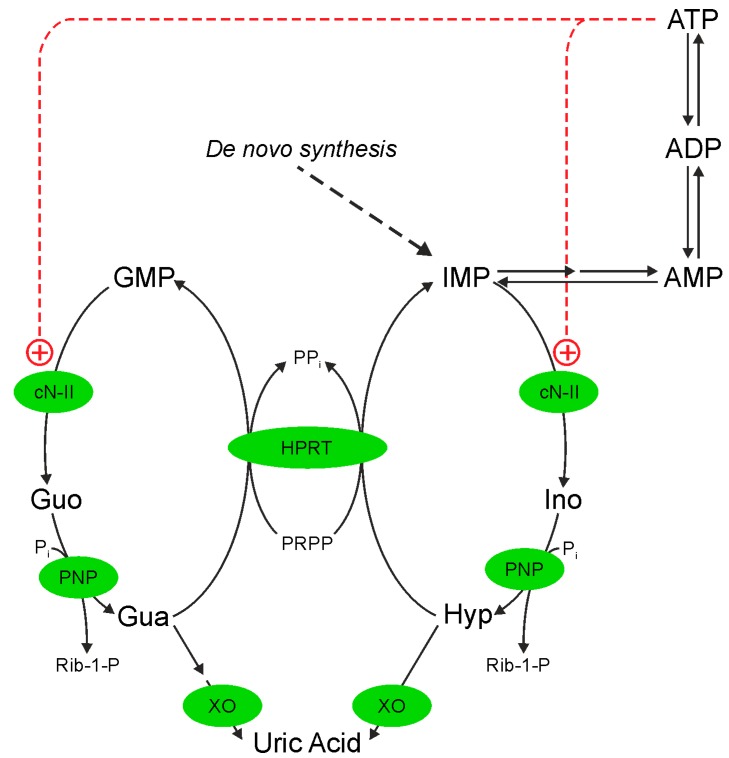
The purine cycle. cN-II: cytosolic 5′-nucleotidase II; PNP: purine nucleoside phosphorylase; HPRT: hypoxanthine guanine phosphoribosyl transferase; XO: xanthine oxidase. The activation of cN-II by ATP is shown (+).

Genomic instability is the principal hallmark of tumor cells and can be a consequence of a dysregulation of nucleotide de novo and/or salvage synthesis or catabolism. A family of 5′-nucleotidases expressed in cytosol or mitochondrial matrix are involved in purine nucleoside monophosphate degradation. Among them, being allosterically regulated, cytosolic 5′-nucleotidase II (cN-II) has been indicated as playing a major role in the intracellular nucleotide and nucleoside homeostasis [1,2]. In fact, in the presence of high energy charge, the enzyme catalyzes the catabolism of excess of neo-synthesized or salvaged inosine monophosphate (IMP), which is eventually converted into uric acid, while allowing for intracellular IMP and adenosine monophosphate (AMP) accumulation in case of low energy charge or adenosine triphosphate (ATP) hydrolysis (see Scheme 1).

cN-II, which is highly specific for IMP and guanosine monophosphate (GMP), is an allosteric enzyme existing in at least three different conformations. A low activity conformation is stabilized by inorganic phosphate and displays high K_M_ for its substrates. A high activity conformation is stabilized by a number of phosphorylated compounds such as diadenosine tetraphosphate, ATP, adenosine diphosphate (ADP), 2,3-bisphosphoglycerate and high energy charge [3,4,5]. Finally, an inactive conformation can be induced by oxidative stress, probably through the formation of a disulphide bridge between C175 and C547 [3]. cN-II is widely expressed and its specific activity is lower than that of other enzymes involved in nucleotide metabolism [6]. Thus, the enzyme appears to catalyze the rate-limiting step in nucleotide catabolism and salvage pathways [6]. The highest cN-II activities have been measured in neoplastic tissues or cells with high nucleic acid turnover [6,7]. Several authors indicated that cN-II is involved in drug resistance in antitumor chemotherapy based on the use of nucleoside analogs, even when the drugs are not substrates of the enzyme, indicating that cN-II may be a general prognostic marker of survival [8,9,10,11]. These observations prompted several research groups to develop cN-II inhibitors that could be applied in chemotherapeutic protocols alone or in combination [12]. As a consequence, a number of cell models in which cN-II was, at some degree, silenced were developed, to unravel the molecular consequences of the enzyme inhibition. In a human astrocytoma cell line (ADF), transitory and partial cN-II silencing prompted apoptosis [13], while partial constitutive cN-II knockdown caused a decrease of cell proliferation [14]. On the other hand, the hyperexpression of cN-II in ADF cells was accompanied by an increase in cell proliferation [14]. Bovine cN-II has been heterologously expressed in *Saccharomyces cerevisiae* [15] which possesses a soluble 5′-nucleotidase, coded by gene *ISN1* [16]. Bovine cN-II and the yeast enzyme (Isn1p) differ for both substrate specificity and regulation. The yeast cells harbouring cN-II displayed, as compared to the control strain, a shorter duplication time and a significant reduction in the nucleoside triphosphate pools with a concomitant decrease in the energy charge [15]. Therefore, in a number of cell models, the specific activity of cN-II appears to be correlated with cell proliferation [6,14,15]. This seems, however, to be cell-specific as similar modifications of cN-II expression in other cell lines not always modified cell proliferation rate [17,18]. Recently we demonstrated that cN-II interacts with NLR family CARD domain-containing protein 4 (Ipaf), opening for this enzyme a new mechanism through which it can modulate cell functions besides altering intracellular nucleotide concentrations [19].

In this paper, using as a model a human lung carcinoma cell line (A549), expressing a cN-II level (approximately 5.5 nmol min^−1^ mg^−1^) higher than the average value measured in a number of different normal tissues (approximately 2 nmol min^−1^ mg^−1^) [6], we mimicked inhibition of cN-II by partially silencing the enzyme. Furthermore, a less active enzyme conformation was stabilized by decreasing energy charge and inducing oxidative stress through incubation with 2-deoxyglucose (dG) in comparable concentration with glucose. We investigated the effect of the modulation of the enzyme activity on nucleotide content, mitochondrial mass, mitochondrial reactive oxygen species (ROS) and mitochondrial membrane potential, protein synthesis and autophagy, migration and proliferative capacity. We found that 50% cN-II silencing in our tumor cell line model gave rise to a more oxidative, less proliferating phenotype thus counteracting some of the cancer features of A549 cells. We also demonstrated that the effects of cN-II silencing are not specific to lung tumor cells, since in human astrocytoma ADF cells a partial constitutive cN-II silencing is followed by a decrease of cell proliferation and a shift toward an oxidative metabolism.

## 2. Results

### 2.1. cN-II Activity and GSH Content

**Figure 1:**
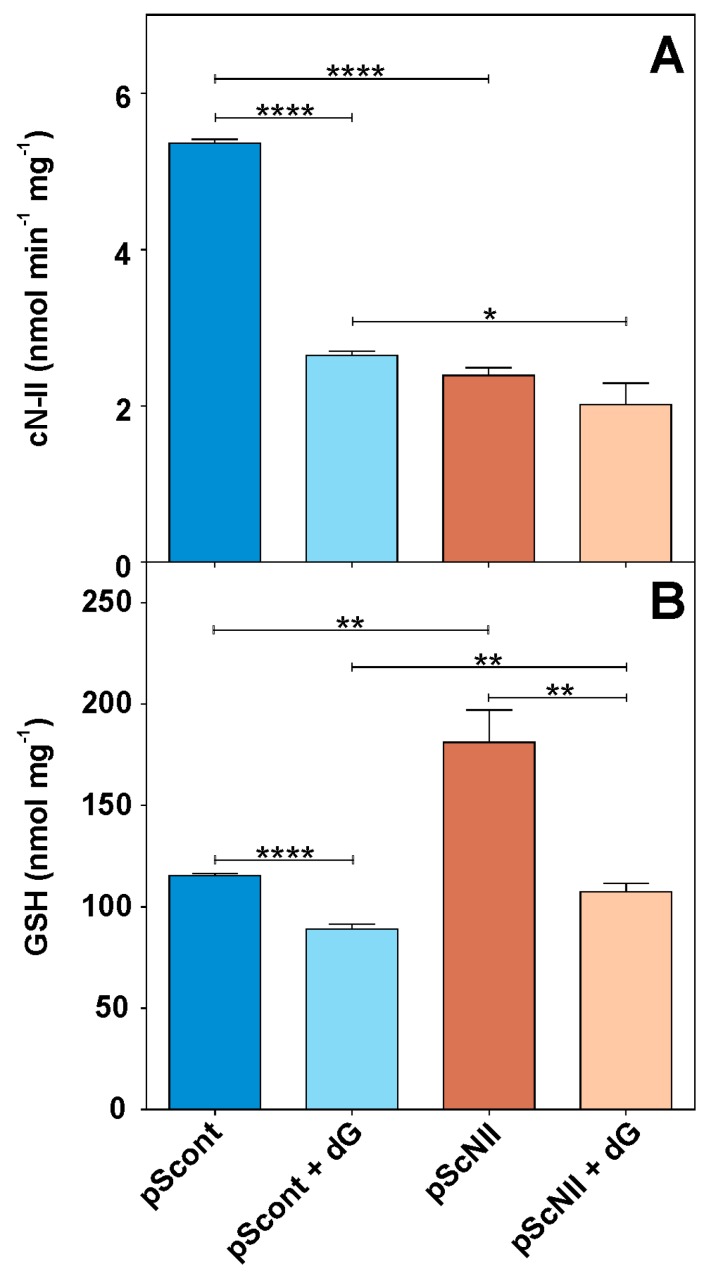
Effect of cN-II silencing on GSH content in A549 cells. (**A**) cN-II activity in pScont and pScNII grown 24 h in the presence or absence of 20 mM dG; (**B**) cellular content of reduced glutathione in the same samples. Results are the mean + SEM of three independent experiments. * *p <* 0.05, ** *p <* 0.01, **** *p <* 0.0001.

In order to test the effect of cN-II inhibition on tumor cell performances, we reduced cN-II activity by silencing it. For this purpose, we utilized human A549 pScont and pScNII cells (stably transfected with non-targeting control shRNA and with cN-II targeting shRNA, respectively), obtained as described by Cividini et al. [19]. In A549-pScNII cells, cN-II activity was only partially silenced being approximately 45% of the parental A549-pScont cells (Figure 1A). Immunoblotting analysis were in line with enzyme activity (Supplementary material Figure S1). Exposure to dG decreased cN-II activity of about 50% in pScont cells, as compared to only approximately 15% in pScNII cells. This result can be due to oxidative damage and might indicate a better antioxidant capacity of pScNII cells. Therefore, we determined the amount of GSH in pScont and pScNII cells incubated with or without dG for 24 h. Figure 1B shows that pScNII cells exhibit a higher content of GSH with respect to control and that incubation with dG causes a decrease of GSH in both cell lines.

### 2.2. Energy Charge and Adenylate Content

**Figure 2:**
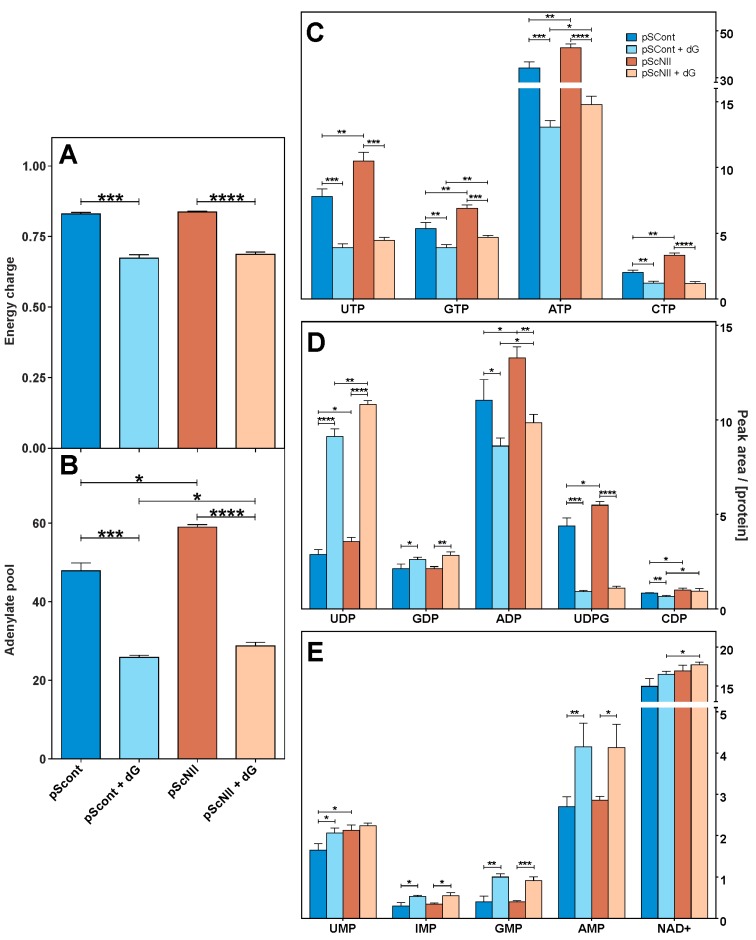
Effect of cN-II silencing on purine and pyrimidine nucleotide content in A549 cells. (**A**) Energy charge and (**B**) adenylate content. Energy charge was calculated on the basis of the capillary electrophoresis analysis. Adenylate pool is expressed as the sum of the peak area of each adenylated compound obtained by capillary electrophoresis; (**C**) triphosphate nucleoside content, (**D**) diphosphate nucleoside content, (**E**) monophosphate nucleoside and NAD^+^ content. Results are the mean + SEM of three independent experiments and are expressed as peak area of each compound normalized on the protein content. * *p <* 0.05, ** *p <* 0.01, *** *p <* 0.001, **** *p <* 0.0001.

Since cN-II participates in the maintenance of purine homeostasis (Scheme 1), the manipulation of its activity is expected to affect the adenylate compound content. A549-pScNII cells contain a significantly higher (about 20%) concentration of adenylyl compounds with a comparable energy charge with respect to pScont cells (Figure 2A,B). Addition of dG in culture media caused a decrease in adenylate content and energy charge that was similar in both cell models. pScNII cells contain a greater amount of triphosphorylated and, to a lesser extent, diphosphorylated purine and pyrimidine compounds, with the exception of guanosine diphosphate (GDP), while no differences were found in nicotinamide adenine dinucleotide (NAD^+^) and nucleoside monophosphates, except for uridine monophosphate (UMP). Exposure to dG caused a decrease of all the triphosphorylated compounds and of ADP, uridine diphospho-glucose (UDPG) and cytidine diphosphate (CDP) at least in pScont cells, while uridine diphosphate (UDP) and GDP increased considerably. Nucleoside monophosphates increased following dG exposure, while NAD^+^ level was significantly affected only in cN-II silenced cells relatively to the control cells (Figure 2C–E).

### 2.3. Cell Proliferation and Viability

**Figure 3:**
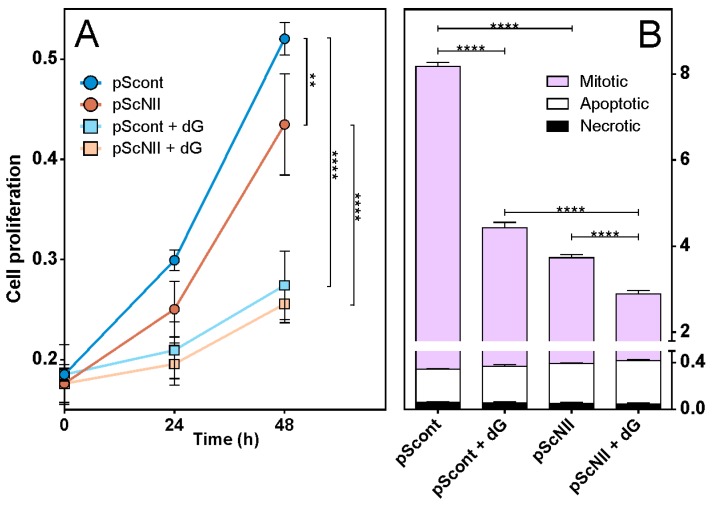
A549 cell proliferation and viability. (**A**) A549 pScont and pScNII growth with or without 20 mM dG was monitored at 24 and 48 h, measuring the absorbance at 596 nm of cells stained with crystal violet. Data are the mean + SEM of two experiments performed in sextuplicate; (**B**) the percentage of mitotic, apoptotic and necrotic cells at 24 h of growth is reported. Results are the mean + SEM of three independent experiments. ** *p <* 0.01, **** *p <* 0.0001. Statistical analysis in A gave the same results after 24 h of culture.

Growth curves of A549-pScont and pScNII cells were monitored both in absence and presence of dG. The analysis demonstrated that at both 24 and 48 h of growth, pScNII cells had a significantly lower proliferative capacity as compared to control cells (Figure 3A). As expected, the addition of dG to the growth medium decreased the proliferative capacity of both cell lines, however, no difference between pScont and pScNII cells was observed. Further analyses indicated that, after 24 h of growth, pScNII showed a lower number of mitotic cells with respect to pScont, thus confirming the data of the growth curve. Furthermore, no remarkable differences were observed in the percentage of apoptotic and necrotic cells in pScNII as compared to pScont, both with and without exposure to dG (Figure 3B).

### 2.4. Enzyme Activities

**Figure 4:**
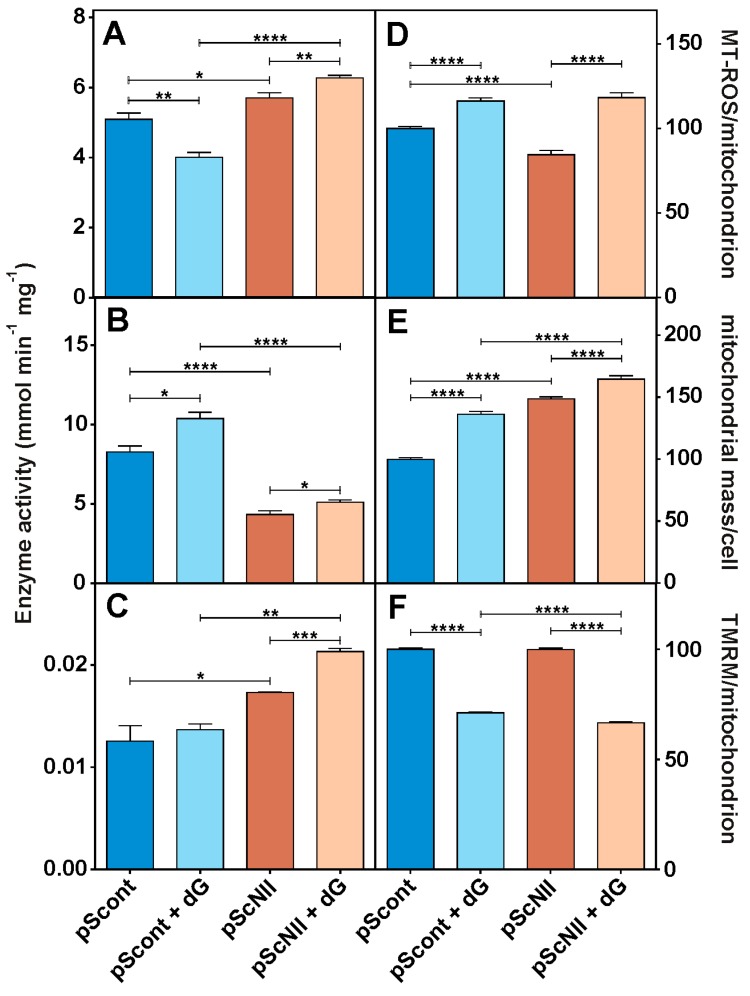
Effect of cN-II silencing on enzyme activities, mitochondrial ROS, mass and membrane potential in A549 cells. (**A**) LDH, (**B**) hexokinase and (**C**) CS were assayed as described in Materials and Methods in cell extracts obtained from cultured pScont and pScNII incubated 24 h with or without 20 mM dG. Each experiment was performed in triplicate and repeated at least three times; (**D**) mitochondrial ROS; (**E**) mitochondrial mass; (**F**) mitochondrial potential. The analysis was performed with confocal microscopy as described in Material and Methods in pScont and pScNII cells incubated for 24 h in the presence or absence of 20 mM dG. Data are the average + SEM of three independent experiments. * *p <* 0.05, ** *p <* 0.01, *** *p <* 0.001, **** *p <* 0.0001.

In order to assess whether cN-II silencing had an effect on glycolytic and oxidative metabolism, we measured the activities of two markers of glycolytic performance [lactate dehydrogenase (LDH) and hexokinase], and one marker of oxidative performance [citrate synthase (CS)]. LDH, hexokinase and CS activities were measured in A549-pScNII and pScont cells incubated with or without dG (Figure 4A–C). cN-II silenced cells display significant higher LDH and CS and lower hexokinase activities with respect to the control. This enzymatic arrangement might be suggestive of a transition from a more glycolytic phenotype in control cells with high cN-II activity, to a more oxidative phenotype in cN-II silenced cells. Addition of dG caused in both cell lines an increase of hexokinase activity which could be compensatory for the inhibition of the glycolytic pathway exerted by dG [20] and, in pScNII cells, an increase of CS, which is in line with the increase of mitochondrial mass (Figure 4E). Exposure to dG causes a decrease of LDH in pScont but, surprisingly, a significant increase in pScNII cells.

### 2.5. Mitochondrial ROS, Membrane Potential and Mass

Figure 4D shows that the mitochondrial ROS are lower in A549-pScNII cells grown in normal conditions. Mitochondrial mass of pScNII cells (Figure 4E) is considerably greater than the mass of pScont cells, while the potential is similar (Figure 4F). This result is compatible with a mitochondrial expansion following cN-II silencing accompanied by the maintenance of the potential and a decrease of ROS level, which can be due to a greater capacity to counteract oxidative stress. Addition of dG causes an increase in mitochondrial ROS and mass accompanied by marks of mitochondrial dysfunctions such as a lower potential. These modifications were similar in both pScont and pScNII cells. Confocal images (Supplementary material Figure S2) clearly demonstrated that cN-II inhibition is followed by an evident increase of mitochondrial fusion, which is even more evident after incubation with dG.

### 2.6. Western Blot Analysis

The metabolic transformation present in A549-pScNII apparently links cN-II activity or expression to a glycolytic high replicative phenotype, while a decrease of cN-II expression or activity apparently is associated with a more oxidative phenotype and a better antioxidant capacity. We then performed immunoblotting analysis to unravel the signaling pathways involved in the cellular transformations that follow cN-II silencing. We measured both total and phosphorylated p53, Akt and AMPK, and the marker of autophagy LC3, in pScont and pScN-II after 24 h of incubation with and without dG.

**Figure 5:**
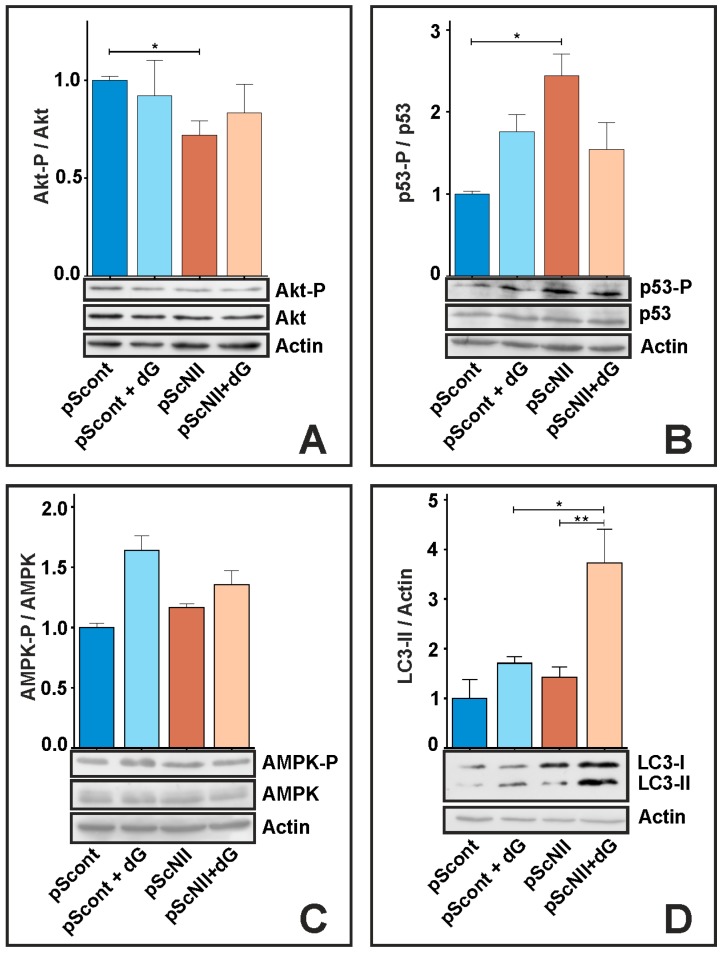
Immunoblotting analysis. Representative images of the immunoblot analysis for (**A**) Akt, (**B**) p53 (**C**) AMPK, and (**D**) LC3 at 24 h of incubation with or without 20 mM dG are shown. β-actin was used as loading control. Densitometry analysis was performed and the ratio (**A**) Akt-P/Akt, (**B**) p53-P/p53, (**C**) AMPK-P/AMPK, and (**D**) LC3-II/actin was calculated. The results are the mean ± SEM of three independent experiments. * *p* < 0.05, ** *p <* 0.01.

Figure 5A shows that the ratio Akt-P/Akt is much lower in pScNII than in pScont cells, and no significant differences were induced by exposure to dG. In pScNII cells, p53 is phosphorylated at a greater extent than in control cells, while after 24 h exposure to dG the ratio p53-P/p53 is not significantly affected (Figure 5B). The ratio AMPK-P/AMPK remained similar between pScont and pScNII cells both with or without exposure to dG (Figure 5C). LC3-I is present at higher extent in pScNII, and a 24 h incubation with dG caused a strong increase of LC3-II, particularly evident in pScNII cells (Figure 5D).

### 2.7. Protein Synthesis

**Figure 6:**
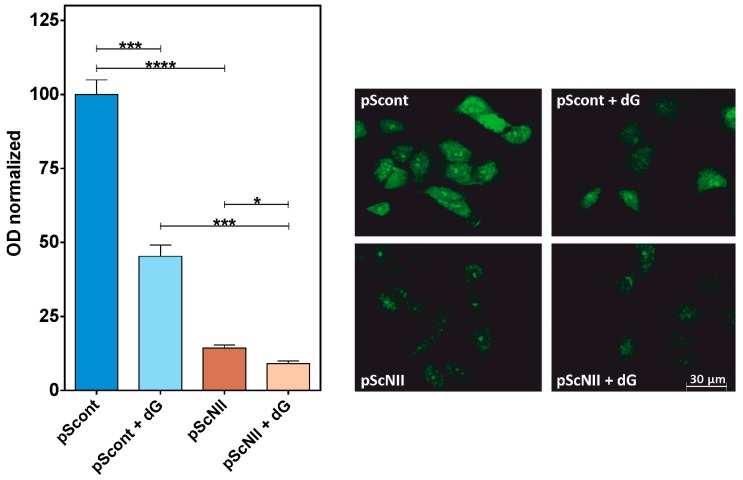
Effect of cN-II silencing on protein synthesis in A549 cells. Newly synthetized proteins were labeled using the Click-iT^®^ HPG Alexa Fluor Protein Synthesis kit (see Methods for details). The incorporation of the methionine analogue HPG was monitored with confocal microscopy. **Left**: optical density (OD) of the fluorescence signal generated by Alexa Fluor 488-azide crosslinked to HPG in A549 cells pScont and pScNII with or without 20 mM dG. Results are the mean + SEM of three independent experiments. * *p <* 0.05, *** *p <* 0.001, **** *p <* 0.0001. **Right**: confocal images of A549 cells after HPG incorporation and Alexa Fluor 488-azide reaction. Calibration bar = 30 µm applies to all micrographs.

The p53-dependent pathway is involved in the regulation of several cellular mechanisms such as proliferation, migration and protein synthesis [21,22,23]. A549-pScont and pScNII were incubated with and without dG and with a methionine analog for 2 h, before determining the rate of protein synthesis by confocal microscopy analysis. Figure 6 shows that the rate of protein synthesis is dramatically lower in cN-II silenced cells compared to control cells, and that incubation with dG further decreases the rate of the methionine analog incorporation in proteins.

### 2.8. Wound Healing Assay

**Figure 7:**
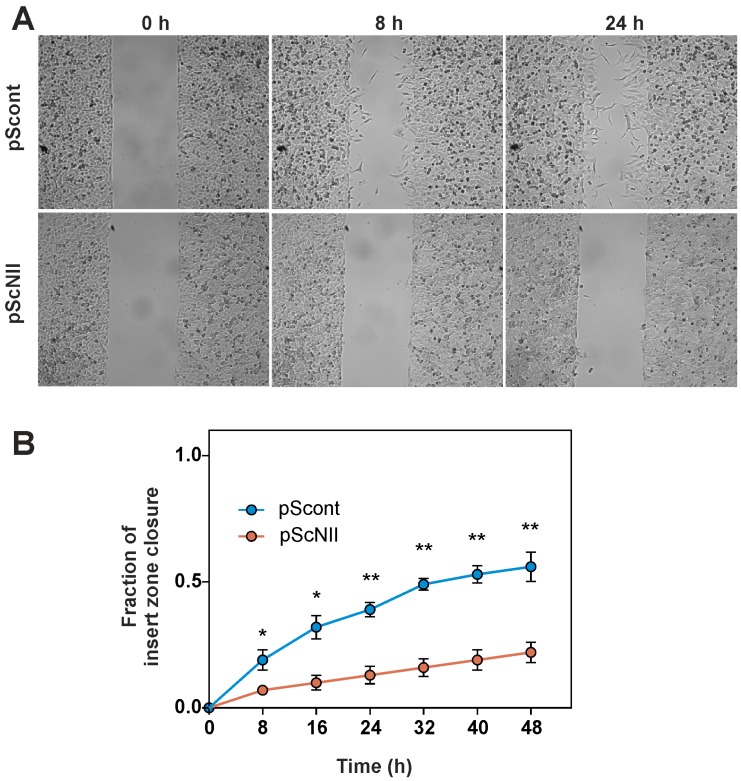
Wound healing assay in A549 cells. The assay was performed as described in Materials and Methods in a medium containing 0.25% FBS with a JuLI Br Live Cell Movie Analyser. (**A**) Representative images captured at 0, 8, 20 h during assay performed on pScont and pScNII cells (wound width is 500 μm); (**B**) percentage of the insert zone closure in the wound edge as function of time. The results are the mean + SEM of three independent experiments. * *p <* 0.05, ** *p <* 0.01.

As mentioned before, p53 is involved in the regulation of cell migration ability [23], therefore we performed a wound healing assay on A549-pScont and pScNII cells. The results revealed that cN-II silencing is followed by a decrease of the migration rate that was significant at all times (Figure 7).

### 2.9. cN-II Silencing in ADF Cells

**Figure 8:**
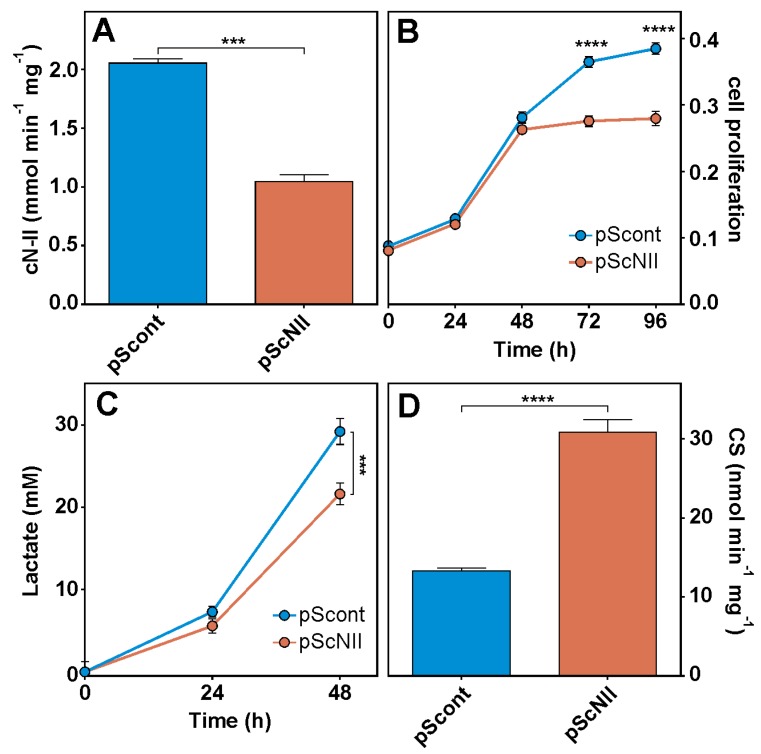
Effect of cN-II silencing in ADF cells. (**A**) cN-II activity in pScont and pScNII; (**B**) ADF pScont and pScNII cell growth; (**C**) lactate level at 24 and 48 h of growth; (**D**) CS activity. The assays were performed as described in Materials and Methods. The results are the mean + SEM of three independent experiments. *** *p <* 0.001, **** *p <* 0.0001.

In order to confirm the results obtained with A549 cells in an additional cell model, we used human ADF-pScont and pScNII cells (stably transfected with non-targeting control shRNA and with cN-II targeting shRNA, respectively) described in [14]. In ADF-pScNII cells, cN-II activity is about 50% that of pScont (Figure 8A), and the growth rate appears considerably lower with respect to control cells (Figure 8B). Furthermore, a decreased level of lactate in medium during growth (Figure 8C) and a doubling of CS activity (Figure 8D) were measured in cN-II-silenced cells compared to control cells.

## 3. Discussion

A549 cells are largely utilized as a model for studies on cancer related metabolism and therapy and as epithelial cell model for drug metabolism [24]. We have previously utilized A549-pScNII cells to demonstrate the interaction between Ipaf and cN-II [19]. We utilized here the same cell model to mimic cN-II inhibition in tumor cells. A partial cN-II silencing in this cell line (residual activity approximately 45% of control), brought about several differential features with respect to control cells. In fact, cN-II silencing is accompanied by an increase of intracellular phosphorylated purine and pyrimidine compounds. It has been previously demonstrated that an increase of cN-II activity usually causes a boost of nucleotide catabolism [15,25]. It is therefore reasonable that cN-II silencing can cause an increase of intracellular triphosphorylated nucleosides; nevertheless, to our knowledge, this is the first time that this effect of cN-II silencing on purine and pyrimidine nucleotide pools is described. We previously demonstrated that in the presence of physiological phosphate concentration and low energy charge (around 0.6), cN-II assumed a conformation with a very high K_M_ for its substrates and low k_cat_ [4]; furthermore, in oxidant conditions, cN-II loses its activity [1]. Therefore, we stabilized the low activity conformation of cN-II lowering the energy charge by incubation with dG, a good inhibitor of both glycolysis and pentose phosphate pathways which also cause oxidative stress [26]. Incubation with dG causes a great decrease of energy charge and adenylate content in both A549 pScNII and pScont cells. Intracellular concentration of GSH is a good marker of cell antioxidant performances [27,28]. In this regard, cN-II silenced cells, which exhibit a GSH content higher than control cells, might have a better protection from oxidative stress. This result suggests that cN-II silencing is followed by an increase of pentose phosphate pathway, generating an increase of reducing power in cells. Our results show that cN-II partial silencing is accompanied also by a decrease of cell growth. The presence of dG further decreased the growth of both A549 pScont and pScNII, while no remarkable effect was observed on apoptotic and necrotic cell number. We previously demonstrated that the heterologous expression of bovine cN-II in *Saccharomyces cerevisiae* caused an increase of cell proliferation [15]. Furthermore, in ADF cells, cN-II silencing was associated with a decrease of cell proliferation, while cN-II hyperexpression caused an increase of cell growth [14]. In the present study, we demonstrate that ADF cell growth in high glucose medium (the same condition adopted for A549) is equally affected by conditional cN-II silencing, thus indicating that the effect exerted by cN-II silencing on cell proliferation is not a peculiarity of A549 cell model. We therefore decided to deepen our knowledge on the effect of cN-II silencing on several cell processes. The decrease of hexokinase activity and the increase of CS in A549 silenced cells indicate for the latter a more active oxidative metabolism with respect to control cells. Confocal microscopy analysis indicated the presence in A549 pScNII cells of a higher mitochondrial mass with lower ROS content, thus confirming an increase of oxidative metabolism and antioxidant defense in silenced cells. A decrease of LDH activity was demonstrated in different cancer models after dG exposure [20], and indeed a decrease of LDH activity was observed in pScont cells, while unexpectedly in A549-pScNII cells, addition of dG causes an increase of LDH activity. Finally, CS activity, which can be considered as a measure of mitochondrial functionality, resulted to be increased in A549-pScNII cells after dG exposure. This is in line with the observation that mitochondrial mass increased as well. Furthermore, the increase of mitochondrial fusion following cN-II inhibition is strongly indicative of a push towards a more oxidative metabolism [29]. Also, in ADF cells, the lower rate of lactate production during growth of cN-II silenced cells with respect to control, and the higher CS activity indicate a relationship between cN-II silencing and a metabolic shift toward a more oxidative phenotype. The capacity to proliferate is strictly linked to the capacity to perform protein synthesis at high rate. The most striking effect of cN-II silencing in A549 cells is a remarkable decrease of the protein synthesis rate that is further decreased following dG incubation. Besides the reduction of cell proliferation, also the migration ability of pScNII cells was remarkably impaired. Overall, our results show that cN-II silencing is accompanied by a change in glucose catabolism pathway, increase of reducing power, mitochondrial expansion, a decrease in the rate of cell growth and mobility and protein synthesis, while autophagy is activated. Since A549 cells express wild type p53 [30], this picture is compatible with the observed activation of the tumor suppressor p53, which can be caused by a mild cell stress leading to a shift of glucose metabolism from fermentation to oxidation [31], increase of oxidant defense [32], Akt dephosphorylation [33], protein synthesis inhibition and authophagy activation [21,22], inhibition of cell growth and mobility [23]. Even though a more complicated explanation cannot be ruled out, the imbalance of purine and pyrimidine compounds in silenced cells reported in the present investigation might well be the origin of the mild stress activating p53. ADF cells, utilized to validate our results, are actually mutated for p53 in the DNA binding sequence, but the mutation cannot prevent the translocation of the protein in the nucleus, and the activation of p53-mediated apoptosis following incubation with proteasome inhibitors and etoposide [34]. Furthermore, transient cN-II silencing in this cell model activated apoptosis [13]. Therefore, it is not surprising that, despite the mutation, also ADF cells were strongly affected by cN-II silencing probably through p53 activation.

Apparently, AMPK does not play a role in the effect exerted by cN-II silencing and dG incubation in A549, probably because LKB1, the principal kinase involved in AMPK activation, is mutated in these cells [35]. On the other hand, it was previously demonstrated that, in LKB1 deficient cells, AMPK can be activated by a different mechanism independent of T172 phosphorylation [36]. Our results are in line with recent data demonstrating that MDA-MB-231 cells silenced for cN-II had better defense against ROS, performed more oxidative metabolism and thus were more sensitive to hypoxia and glucose deprivation than control cells [37].

In conclusion, cN-II besides its ability in regulating the intracellular purine nucleotide concentrations, is also involved in the regulation of fundamental cellular functions such as protein synthesis and cell proliferation. In fact, a 50% decrease of cN-II specific activity obtained by constitutive silencing, is sufficient to induce a phenotype remarkably distinct from that of control cells. A phenotype in which the metabolic and behavioral characteristics associated to tumor cells are mitigated, becoming more similar to the features of normal cells. Activation of tumor-suppressor p53, probably caused by a purine and pyrimidine imbalance, appears to mediate all the effects described in this paper. These results might help to cast the bases of new therapeutic approaches targeting cN-II active conformation.

## 4. Materials and Methods

### 4.1. Materials

Protease inhibitor cocktail, ATP, 2,3-bisphosphoglyceric acid, ethylenediaminetetraacetic acid (EDTA), d-glucose, phenylmethyl sulphonyl fluoride (PMSF), 5,5′-dithiobis(2-nitrobenzoic acid) (DTNB), sodium fluoride, sodium orthovanadate, β-glycerophosphate, sodium pyrophosphate, dimethylsulfoxide (DMSO), percloric acid, glucose-6-phosphate dehydrogenase (G6PDH) (EC1.1.1.49), pyruvate, acetyl CoA, oxaloacetate, NADH, NADP+, crystal violet, Hoechst 33258, ABTS(2,20-azino-di-[3-ethylbenzothiazoline-sulfonate]), l-lactate oxidase, horseradish peroxidase were from Sigma (Milano, Italy); dG was from Carbosynth Ltd. (Berkshire, UK); [8-^14^C] Inosine (6000 dpm/nmol) was from Moravek Biochemicals and Radiochemicals (Brea, CA, USA); Dulbecco’s modified Eagle’s medium (high glucose) (DMEM), RPMI medium, penicillin, streptomycin, foetal bovine serum (FBS), glutamine and trypsin were from Euroclone (Pero, Milan, Italy) MitoTracker Green (MT-Green), tetramethylrhodamine methyl ester (TMRM) and MitoTracker Red CM-H2XROS (MT-ROS) from Molecular Probes, Invitrogen (San Giuliano Milanese, Milan, Italy). Click-iTTM HPG Alexa Fluor 488 Protein Synthesis Assay Kit was from Thermo Fisher Scientific (Walthman, MA, USA); Chemiluminescence Detection System was from Millipore, (Burlington, Massachusetts, USA); primary antibodies specific for Akt(pS473) (NBP1-69923) and AMPK(pThr172) (NBP1-74502) were from Novus Biologicals (Littleton, Colorado, USA); primary antibodies specific for Akt(pan) (#2920), AMPKα1 (#2795), p53(pSer15) (#9286), p53 (#2524), LC3B (#3868), β-Actin (#8457), and HRP-linked anti-mouse (#7076) and anti-rabbit IgG (#7074) were from Cell Signaling (Danvers, Massachusetts, USA); primary antiboby specific for NT5C2 (WH0022978M2) was from Sigma (Milano, Italy). A549 and ADF cell lines were purchased from ATCC (ATCC^®^ CCL-185TM) and routinely tested for *Mycoplasma* contamination by PCR. All other chemicals were of reagent grade.

### 4.2. Cell Culture and Cell Proliferation Assay

Human A549 cells were cultured in DMEM high glucose (25 mM) supplemented with 10% FBS, 1% glutamine, 100 U/mL penicillin and 100 U/mL streptomycin. Human ADF cells were cultured in RPMI supplemented with 25 mM glucose, 10% FBS, 100 U/mL penicillin and 100 U/mL streptomycin. The cells were cultured in an incubator with 5% CO_2_ at 37 °C. Cell proliferation assay was performed by the crystal violet staining method [38]. Briefly, 12 h before treatment, three 24 multi-well tissue culture plates were seeded with 0.6 mL of culture medium containing 20,000 cells/well. When indicated, cells were exposed to 20 mM dG and after 0, 24 and 48 h of incubation, the medium was removed and the cells were stained with 0.1% crystal violet solution in methanol for 30 min at 37 °C under gentle shaking. Then, the plates were carefully washed three times in distilled water and dried. Acetic acid (0.6 mL, 10%) was added to the wells and kept 15 min at room temperature under gentle shaking. One hundred microliters from each well were transferred into a 96 multi-well for the quantitative analysis by absorbance measurements at 596 nm in an automatic ultra microplate reader EL 808 Bio-Tek Instruments. Inc. (Winooski, Vermont, USA).

### 4.3. High Performance Capillary Electrophoresis (HPCE) Analysis

Cells were plated at a density of 3.3 × 10^4^/cm^2^ in 10 mL medium in 100 mm diameter plates. After 12 h, medium was withdrawn and replaced either with 7 mL of the same medium (control samples, 3 plates for each cell type) or with 7 mL of medium containing 20 mM dG (3 plates for each cell type). After 24 h, plates were washed with phosphate buffer saline (PBS) and trypsinized cells were rapidly separated from medium by centrifugation (400× *g* for 5 min) and resuspended in 170 μL 1 M trichloroacetic acid (TCA). To avoid degradation of nucleotides, samples were treated as previously described [1,39]. The protein content of the pellets previously precipitated with TCA was determined using a modified Lowry method [40]. The HPCE analysis was performed on supernatants treated with ethyl ether to remove TCA. All the experiments were performed using an Agilent Capillary Electrophoresis System (Santa Clara, California, USA) equipped with a diode array detector as previously described [15]. All samples were loaded by a low-pressure injection (34 mbar, 15 s); these conditions ensured that the amount loaded was lower than 1% of the total capillary volume. The elution profile was followed at 254 nm.

### 4.4. Reduced Glutathione Assay

Reduced glutathione (GSH) level was determined following Ellman method with some modifications [41]. Cells prepared as described for the HPCE analysis were washed with PBS, trypsinized and resuspended in 400 µL 100 mM Tris-HCl pH 7.4 in the presence of protease inhibitors. Crude extracts were obtained by three freeze/thaw cycles followed by centrifugation at 10,000× *g* for 40 min at 4 °C. The protein content of the supernatant was determined following the method described by Bradford [42]. Sixty-three µL of the supernatant was added to 7 µL of 6 M perchloric acid, strongly shacked and centrifuged 5 min at 10,000× *g*. For the determination of GSH content, 5 µL of deproteinized extract was added to a solution of 2 mM DTNB dissolved in 1% sodium citrate, 250 µM EDTA, 250 mM sodium phosphate buffer pH 7.4 in a total volume of 700 µL. The absorbance at 412 nm was measured using a Beckman spectrophotometer. The GSH levels were calculated using an extinction coefficient of 13,600 M^−1^ cm^−1^.

### 4.5. Enzymatic Assays

Cells prepared as described for the HPCE analysis were washed with PBS, trypsinized and resuspended in 400 µL 100 mM Tris-HCl pH 7.4 in the presence of protease inhibitors cocktail, 10 mM NaF, 30 mM β-glycerophosphate and 10 mM Na-pyrophosphate as phosphatase inhibitors. Crude extracts were obtained by three freeze/thaw cycles followed by centrifugation at 10,000× *g* for 40 min at 4 °C; supernatant was collected and protein content determined.

Hexokinase activity was measured following the method described by Gerber et al. [43] with some modifications. The assay was carried out by continuous spectrophotometric monitoring the absorbance at 340 nm at 30 °C (extinction coefficient of 6220 M^−1^·cm^−1^). The enzyme assay mixture contained: 50 mM Tris-Cl pH 7.4, 120 mM d-glucose, 10 mM MgCl_2_, 0.6 mM ATP, 0.25 mM NADP^+^, 0.25 EU G6PDH and 300 µg of crude extract.

LDH assay was performed spectrophotometrically monitoring the change in absorbance at 340 nm at 37 °C [44]. The enzyme assay mixture contained 50 mM Tris-Cl pH 7.4, 3 mM pyruvate, 0.1 mM NADH and 3 µg of crude extract.

CN-II phosphotransferase activity was assayed following the method previously described [45].

CS assay was performed according to Eigentler et al. [46] with some modifications. The assay was carried out by continuous spectrophotometric monitoring of the change in absorbance of DTNB at 412 nm at 30 °C (extinction coefficient of 13,600 M^−1^·cm^−1^). The enzyme assay mixture contained 80 mM Tris-Cl pH 8.3, 0.5 mM acetyl-CoA, 5 mM oxaloacetate, 10 mM DTNB and 30 µg of crude extract.

### 4.6. Quantification of Mitochondrial Mass, ROS and Membrane Potential

Cells were seeded at a density of 5000 cells/cm^2^ and grown on 13 mm diameter glass coverslips in 24-well plates. After 12 h, medium was withdrawn and replaced with fresh medium with or without 20 mM dG. After 24 h incubation, coverslips with living cells were mounted in a glass chamber on the stage of a laser scanning confocal microscope (TCS-NT Leica, Nussloch, Germany) for imaging. For assessment of mitochondrial mass and ROS level, cells were loaded for 30 min at 37 °C in 5% CO_2_ with 2 nM MT-Green and 2 nM MT-ROS. MT-Green was used to determine the whole mitochondrial mass, as it accumulates in the mitochondrial matrix regardless of transmembrane potential. MT-ROS was used to measure the mitochondrial ROS level. MT-ROS is a reduced, non-fluorescent form of the MitoTracker Red probe, it is sequestered by mitochondria and oxidized by ROS to a red fluorescent compound in actively respiring cells. The double labelling of mitochondria with MT-ROS and MT-Green allowed the normalization to the total mitochondrial mass of the ROS variation (*F*/*F_0_*) after a given cell culture condition. MT-Green probe was excited with the 488 laser line and the emission was collected at 516 ± 10 nm. MT-ROS probe was excited with the 543 laser line and the emission was collected at 600 ± 10 nm. The mitochondrial membrane potential was assessed by staining A549 cells with the fluorescent cationic probe TMRM. Cells were loaded for 30 min at 37 °C in 5% CO_2_ with 5 nM TMRM which distributes into the mitochondrial matrix of active mitochondria following the electrochemical gradient. TMRM was excited with the 543 laser line and emission was collected at 600 ± 10 nm. For confocal imaging, laser power was kept at 10% of the maximal power to avoid cell photo-damage and photo-bleaching of fluorescent probes. Images were acquired at 1024 × 1024 pixel resolution and averaged 4 times to improve the signal/noise ratio. Oil immersion objectives 40× (1.2 NA) or 63× (1.4 NA) (Leica, Nussloch, Germany) were used. The analysis of confocal images was made offline using both the image analysis software MetaMorph 5.0 (Universal Imaging, West Chester, PA, USA) and MatLab routines (The MathWorks, Natick, MA, USA) adapted by our laboratory to cell density and mitochondrial fluorescence evaluation [47]. For each field both fluorescent and transmitted light images were acquired on separate photomultipliers and merged in offline analysis.

### 4.7. Analysis of Mitosis, Apoptosis and Necrosis Incidence

Cells prepared as described for the quantification of mitochondrial mass, were fixed with 2% paraformaldehyde in cold PBS and stained with 1 μg/mL Hoechst dye 33258 for 30 min at room temperature. Cells were visualized using a fluorescence microscope (Carl Zeiss Axiovert 35, Oberkochen, Germany) at 20× magnification. On the basis of morphological criteria, cells were assigned to three groups as follows: small round cells with condensed chromosomes aligned at the middle of the cell (metaphase) as well as dividing cells (late anaphase and telophase) were considered as mitotic, collapsed cells with fragmented nuclei and chromatin condensed in the typical multiple bodies were considered as apoptotic, swollen and dark cells were considered as necrotic. The incidence of mitosis, apoptosis and necrosis was evaluated by counting 30 microscopic fields for each treatment (field area 1 mm^2^, each containing an average of 500 cells).

### 4.8. Western Blotting

Cells, prepared as described for the HPCE analysis, after 24 h of treatment were washed with 1 mL cold PBS in the presence of protease inhibitor cocktail, supplemented with 1 mM PMSF, 1 mM sodium orthovanadate, 5 mM sodium pyrophosphate, 20 mM β-glycerophosphate. PBS containing protease inhibitors was then removed, cells scraped off and collected. Plates were then washed with 1 mL cold PBS containing protease inhibitors and remaining cells collected and added to the previous ones, then centrifuged at 700× *g* for 5 min at 4 °C. Supernatants were discarded and 200 µL of lysis buffer containing 150 mM NaCl, 50 mM NaF, 0.5 mM EDTA pH 8.0, 0.5 % Triton X-100, 1 mM PMSF, 1 mM sodium orthovanadate, 5 mM sodium pyrophosphate, 20 mM β-glycerophosphate in 25 mM Tris-Cl pH 7.4, was added. Vials with cells and lysis buffer were then strongly shacked for 1 min and kept on ice for 10 min prior to centrifugation at 10,000× *g* for 40 min at 4 °C. Cellular lysates were collected and concentration of protein extracts was determined. Protein samples (40 µg each lane), were resolved by 12% SDS-PAGE (LC3B was resolved by 15 % SDS-PAGE) at 200 V for 45 min and transferred onto polyvinylidene fluoride (PVDF) membranes at 90 V for 1 hour using Bio-Rad transfer system. Membranes were blocked with TBSTa (50 mM Tris-HCl pH 7.5 supplemented with 150 mM NaCl, 0.1% (*v*/*v*) Tween-20) and 5% (*w*/*v*) bovine serum albumin for or 5% (*w*/*v*) skim milk powder (TBSTb). Membranes were incubated with primary antibody overnight at 4 °C then secondary antibody for 1 hour at room temperature before visualizing chemiluminescence of protein bands using Chemiluminescence Detection System. Primary antibodies specific for Akt (pS473) 1:1000 in TBSTa, AMPK (pThr172) 1:500 in TBSTa, p53 (pSer15) 1:500 in TBSTb, p53 1:500 in TBSTb, LC3B 1:1000 in TBSTa, Akt (pan) 1:2000 in TBSTb, AMPKα1 1:500 in TBSTa, NT5C2 1:1000 in TBSTa, and HRP-linked secondary antibodies anti-mouse and anti-rabbit IgG 1:3000 in TBSTb were used. Relative abundance of proteins was determined using Bio Rad Image Lab version 6 software.

### 4.9. Quantification of Protein Synthesis and Lactate Concentration

To detect newly synthesized proteins the Click-iT^TM^ HPG Alexa Fluor 488 Protein Synthesis Assay Kit was used. The assay is based on the incorporation of L-homopropargylglycine (HPG) into newly synthetized proteins. The kit was used according to manufacturer’s instructions. Briefly, cells prepared as described for the quantification of mitochondrial mass, were incubated in methionine-free DMEM medium for 1 hour to deplete the endogenous methionine. Then this medium was removed and replaced with methionine-free DMEM containing 2 mM HPG for 2 h at 37 °C, 5% CO_2_ to allow the incorporation of HPG into nascent proteins. After incubation, cells were washed in standard DMEM to remove the excess amount of HPG, fixed with 2% paraformaldehyde and permeabilized with 0.1% Triton X-100 for 15 min at room temperature, then cells were washed three times for 10 min each at room temperature with PBS. The HPG incorporated in newly synthesized proteins was crosslinked to Alexa Fluor^TM^ 488 azide by the chemoselective ligation between the alkyne moiety of HPG and the azide contained in the Alexa Fluor^TM^ 488, using the Click-iT reaction buffer (30 min incubation at room temperature, protected from light). The HPG incorporation was quantified by monitoring the intensity of Alexa Fluor^TM^ 488 fluorescence in images captured with confocal microscopy and analysed off-line. Cells boundaries were determined using MatLab software (The MathWorks, Natick, MA, USA) and the fluorescence intensity in each cell was defined as the mean intensity of pixels included inside the cell boundaries after the background subtraction. Lactate concentration in growth media was assayed as described by Liaud et al. [48].

### 4.10. Wound Healing Assay

pScont and pScNII were seeded (180,000 cells/cm^2^) in Culture-Inserts 2 wells. After 12 h to allow cell attachment, the insert was gently removed. Thereafter, the medium was discarded and, after two washing with PBS, replaced by DMEM containing 0.25% FBS. Cells were allowed to migrate for 48 h. Images were collected and the percentage of the insert zone closure in the wound edge was calculated by JuLI Br Live Cell Movie Analyser (NanoEnTek USA Inc., Waltham, MA, USA).

### 4.11. Statistical Analysis

All data are reported as the mean ± SEM. Significant differences among groups were determined using the unpaired Student’s *t*-test or the One-Way analysis of variance (ANOVA) followed by Tukey multiple comparison test with GraphPad InStat version 4.0 (GraphPad Software, San Diego, CA, USA) or Origin 8 (OriginLab Corporation, Northampton, MA, USA). A *p*-value < 0.05 was considered to indicate statistical significance.

## Supplementary Materials

Supplementary materials can be found at http://www.mdpi.com/1422-0067/19/7/2115/s1.

## Abbreviations

A549	human lung carcinoma cell line
ADF	human astrocytoma cell line
ADP	adenosine diphosphate
Akt	protein-kinase B
AMP	adenosine monophosphate
AMPK	AMP-dependent protein kinase
CDP	cytidine diphosphate
cN-II	cytosolic 5′-nucleotidase II
CS	citrate synthase
dG	2-deoxyglucose
DMEM	Dulbecco’s modified Eagle’s medium
DMSO	dimethylsulfoxide
DTNB	5,5′-dithiobis(2-nitrobenzoic acid)
EDTA	ethylenediaminetetraacetic acid
FBS	foetal bovine serum
GMP	guanosine monophosphate
GDP	guanosine diphosphate
G6PDH	glucose-6-phosphate dehydrogenase
GSH	reduced glutathione
HPCE	High Performance Capillary Electrophoresis
HPG	L-homopropargylglycine
IMP	inosine monophosphate
LDH	lactic dehydrogenase
NAD	nicotinamide adenine dinucleotide
MT-Green	MitoTracker Green
MT-ROS	MitoTracker Red CM-H_2_XROS
PBS	phosphate-buffered saline
PMSF	phenylmethyl sulphonyl fluoride
PVDF	polyvinylidene fluoride
TBS	TRIS-buffered saline
TCA	trichloroacetic acid
TMRM	tetramethylrhodamine methyl ester
UMP	uridine monophosphate
UDP	uridine diphosphate
UDPG	uridine diphospho-glucose
