# Supplementary material for: Cytosolic 5′-Nucleotidase II Silencing in a Human Lung Carcinoma Cell Line Opposes Cancer Phenotype with a Concomitant Increase in p53 Phosphorylation
Source: Int J Mol Sci. 2018 Jul 20;19(7):2115. doi: 10.3390/ijms19072115 (PMC6073589; doi:10.3390/ijms19072115)
Supplement: Supplementary file 1 [file ijms-19-02115-s001.pdf]

Supplementary Materials: Cytosolic 5′-Nucleotidase II Silencing in a Human Lung Carcinoma Cell Line Opposes Cancer Phenotype with a Concomitant Increase in p53 Phosphorylation

Rossana Pesi, Edoardo Petrotto, Laura Colombaioni, Simone Allegrini, Mercedes Garcia-Gil, Marcella Camici, Lars Petter Jordheim and Maria Grazia Tozzi

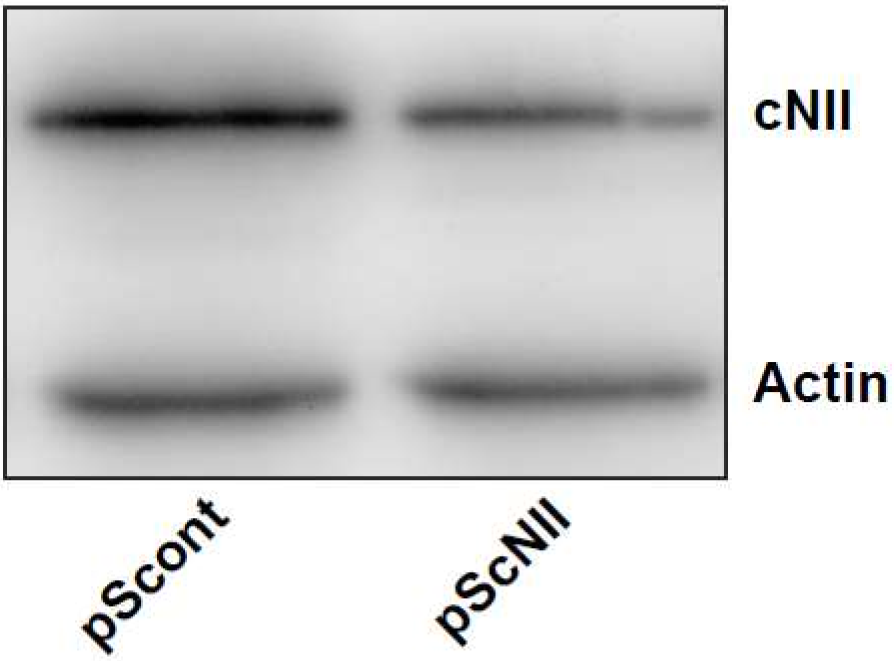

Figure S1. cN-II expression in A549 cells stably transfected with pScont or pScNII. The immunoblot was performed on cell extracts from transfected cells and show the decreased cN-II expression in pScNII transfected cells.

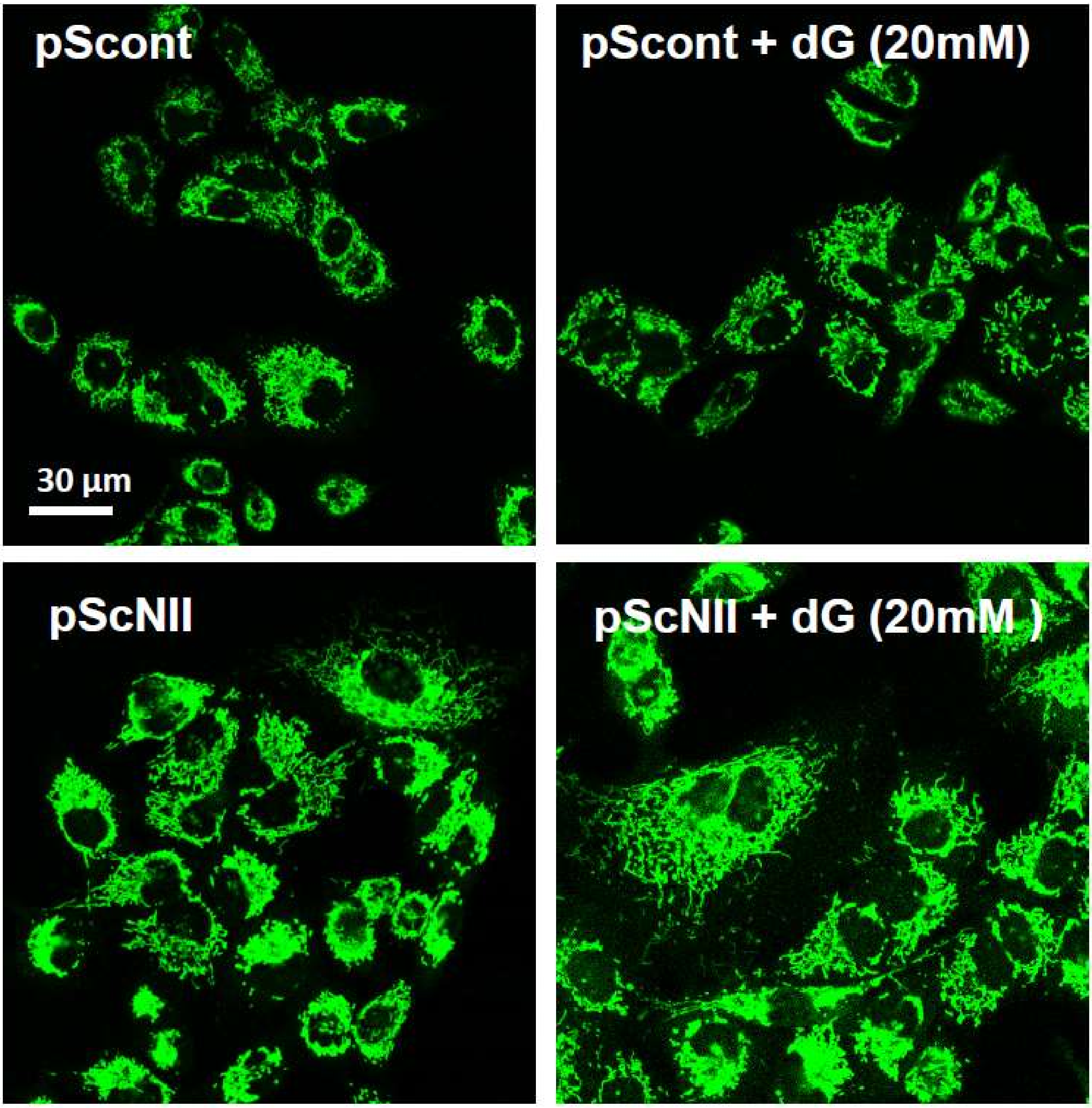

Figure S2. Rearrangements of the mitochondrial network induced by cN-II silencing and by 20 mM dG. Representative images of control (pScont) and cN-II silenced (pScNII) A549 cells with or without 20 mM dG. Living mitochondria were visualized by confocal microscopy after the loading of the fluorescent probe Mitotracker Green (see Methods for details). Calibration bar = 30 µm applies to all images.
